# *Helicobacter pylori* Infection and Psoriasis: A Systematic Review and Meta-Analysis

**DOI:** 10.3390/medicina55100645

**Published:** 2019-09-26

**Authors:** Mingyang Yu, Rongguang Zhang, Peng Ni, Shuaiyin Chen, Guangcai Duan

**Affiliations:** Department of Epidemiology, College of Public Health, Zhengzhou University, Zhengzhou 450001, China; yumingyang2019@126.com (M.Y.); nipeng17@163.com (P.N.); sychen@zzu.edu.cn (S.C.); gcduan@zzu.edu.cn (G.D.)

**Keywords:** *Helicobacter pylori*, infection, psoriasis

## Abstract

*Background and Objectives:* To perform a systematic review and meta-analysis with the aim of determining the relationship between *H. pylori* infection and psoriasis. *Methods*: Pubmed, Embase, China National Knowledge Infrastructure (CNKI), and Web of Science were searched for articles published up to July, 2019. Review Manager 5.3 and Stata 12.0 were used for statistical analyses. *Results*: The initial database search resulted in 204 articles. Through exclusion and screening, 11 studies involving a total of 1741 participants were finally included in this meta-analysis. The odds ratio (OR) of *H. pylori* infection rate in the psoriasis group was significantly higher than that in the control group (OR = 1.19, 95% CI 1.15–2.52, *P* = 0.008). Subgroup analysis showed that no significant difference was detected between the Asia group and the Europe group. As for the methods of *H. pylori* detection, a statistically significant increase of *H. pylori* infection in the IgG ELISA test group was detected, compared with the urea breath test group. In addition, analysis based on the severity of psoriasis showed a statistically significant increase of *H. pylori* infection in moderate and severe psoriasis patients (OR = 2.27; 95% CI: 1.42–3.63, *I*^2^ = 27%), but not in the mild psoriasis patients (OR = 1.10; 95% CI: 0.79–1.54, *I*^2^ = 0%). *Conclusion*: *H. pylori* infection is associated with psoriasis, and psoriasis patients with *H. pylori* infection have higher Psoriasis Area and Severity Index (PASI) scores. The findings are of considerable significance for the clinical practices.

## 1. Introduction

*Helicobacter pylori* is a microaerophilic gram-negative bacterium that naturally colonizes human gastric mucosa. *H. pylori* infection is a global health problem. In some developing countries, the rate of *H. pylori* infection can reach 80–90% [1,2]. It is well known that *H. pylori* infection is closely related to various gastrointestinal diseases, such as duodenal ulcer and gastric cancer [3]. In recent years, the relationship between *H. pylori* infection and extragastrointestinal diseases has attracted the attention of many researchers. It has been reported that *H. pylori* infection is associated with esophageal diseases, dementia, autoimmune skin disease, and so on [4,5,6]. In addition, studies suggested a relationship between *H. pylori* infection and certain skin diseases, which might lead to novel therapeutic strategies for these diseases [7,8,9].

Psoriasis is a common chronic cutaneous disease which seriously affects the life quality of patients. The main clinical manifestations are erythema, papules, and scales [10]. Lesions usually occur in the extensor aspects of elbows and knees but can appear anywhere on the body. The pathogenesis of psoriasis is still unclear. Researchers believe that it is related to genetics, infection, immune abnormalities, and endocrine factors [11]. In terms of treatment, there is currently no cure for psoriasis, however, appropriate symptomatic treatment can control the symptoms [12]. In recent years, researchers have found that *H. pylori* infection is associated with the pathogenesis of psoriasis. Although several studies have shown that treatments for *H. pylori* infection could alleviate the clinical symptoms of psoriasis, some studies have produced controversial results [13,14,15].

Thus, this meta-analysis was conducted to estimate the relationship between *H. pylori* infection and psoriasis, aiming to provide more reliable evidences for clinical psoriasis treatment decision.

## 2. Materials and Methods

### 2.1. Search Strategy

We systematically searched Pubmed, Web of Science, Embase, and China National Knowledge Infrastructure (CNKI) for all the relevant studies investigating the association between *H. pylori* infection and psoriasis published up to July 2019. There were no restrictions on language or publication type. The following search terms were used: (psoriases or psoriasis) and (*H. pylori* or *Helicobacter pylori*). The complete search strategy was included in Table S1. Reference lists of identified articles were also manually searched for other relevant articles.

### 2.2. Selection Criteria

Two independent reviewers reviewed the results of the preliminary searches. Studies were included if they (1) were cohort studies, cross-sectional studies, or case–control studies that assessed the relationship between psoriasis and *H. pylori* infection; (2) compared at least two groups, including (a) patients with any type of psoriasis diagnosed by clinical manifestations or pathology and (b) participants without psoriasis recruited from hospitals or communities; (3) the full text was available; (4) reported the data of the *H. pylori* infection rates in the participants with or without psoriasis; (5) diagnosed *H. pylori* infection by histology, IgG ELISA test, urea breath test, or stool antigen test.

Studies were excluded if they (1) were conference summaries, case reports, reviews, letters, or comments; (2) included the study participants with a specific disease (e.g., cardiovascular diseases, kidney diseases, etc.) or patients who had taken antibiotics, proton pump inhibitors, antacids, or glucocorticoids the preceding 2 weeks; (3) had incomplete or missing information.

In case of duplicate reports, or studies clearly reported results from the same study population. We chose the latest or most complete one.

### 2.3. Data Extraction and Quality Assessment

Two reviewers (Yu and Ni) used the standardized data abstraction sheets to extract the data independently. Disagreements were resolved by consulting a third reviewer (Zh). The following information from each article was extracted: the first author’s name, published year, location, study design, baseline features of the involved participants, the Psoriasis Area and Severity Index of the patients, and outcomes of *H. pylori* infection test. The authors were contacted if we could not obtain the required information in original articles.

The Newcastle–Ottawa Scale (NOS) [16] was used to estimate the quality of each included study. The scale scores ranged from 0 to 9. We considered the higher the score, the better the quality of the study. The NOS score of 0–3 was considered poor, while 4–6 was fair and 7–10 was good quality. Two independent reviewers completed the entire quality assessment. Consensus on quality assessment was reached via discussion.

### 2.4. Statistical Analysis

This meta-analysis was conducted by Review Manager 5.3 and Stata 12.0. We analyzed the outcomes that included incidence of *H. pylori* infection between psoriasis and control groups and expressed them as an odds ratio (OR) with a 95% confidence interval (CI). *P* < 0.05 was considered as statistically significant. Heterogeneity among studies was evaluated via chi-square tests and the inconsistency statistic. The levels of heterogeneity assessed by *I^2^* were as follows: 0–25% meant homogeneity; 25–50% meant low heterogeneity; 50–75% meant moderate heterogeneity, and >75% meant high heterogeneity [17]. The random-effects model was used when *I^2^* > 50%, otherwise, the fixed-effects model was adopted. Subgroup analyses were conducted by locations and the methods of *H. pylori* detection. In addition, potential publication bias was assessed by Begg’s and Egger’s tests. *P* values < 0.05 were considered statistically significant.

## 3. Results

### 3.1. Literature Search and Study Characteristics

**Figure 1:**
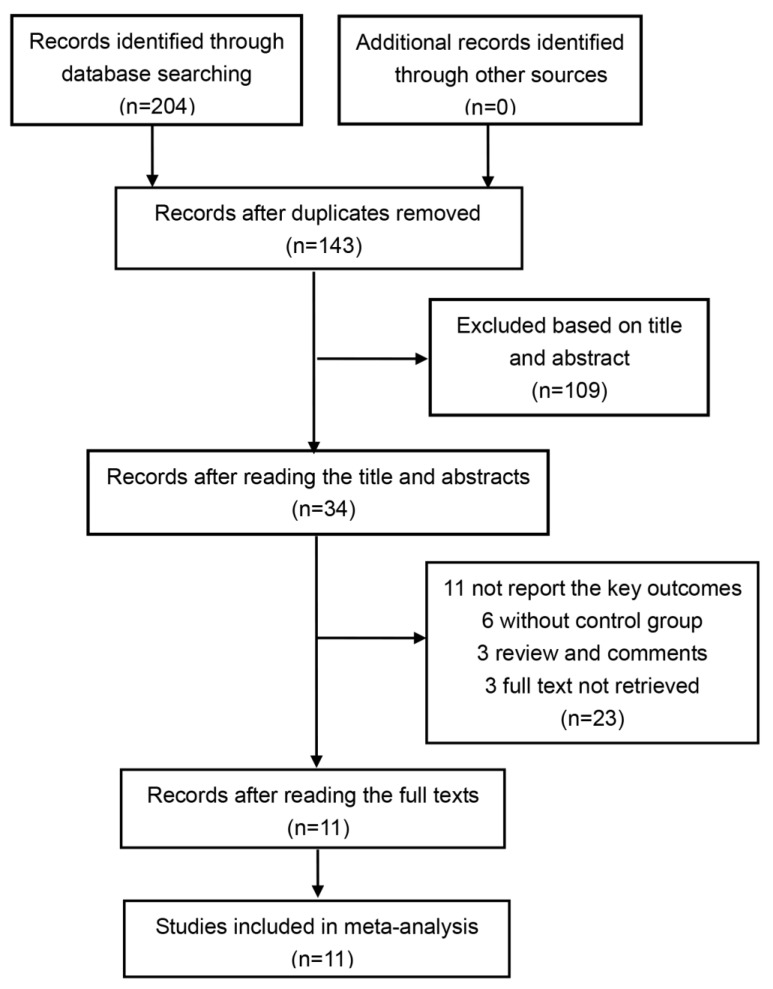
Flowchart of the study selection.

The initial database search generated 204 articles, 61 of which were excluded because of duplicates. After reading the title and abstract, 109 records were excluded via the initial screening. We then screened the whole 34 articles and finally included 11 studies for analysis (1741 participants) [18,19,20,21,22,23,24,25,26,27,28]. Figure 1 details the selection and exclusion process.

Among the 11 studies included in the meta-analysis, three were cohort study [19,21,27], four were cross-sectional study [20,23,24,25] and four were case–control study [18,22,26,28]. The publication year of the involved studies varied from 2000 [18] to 2017 [27]. The number of the participants in each study ranged from 40 [20] to 450 [21]. Among all the studies, five were from Asia [21,24,25,26,28], four from Europe [18,19,22,23], one from South America [27], and one from Africa [20]. As for the detection method of *H. pylori*, five studies used the IgG ELISA test [20,23,24,26,27] and five studies used the urea breath test [18,19,22,25,28], while Onsun N et al. [21] used the stool antigen test. All of the included studies were graded as moderate and good quality. Table 1 details the characteristics of each included study.

### 3.2. Psoriasis and H. pylori Infection Rates

**Figure 2:**
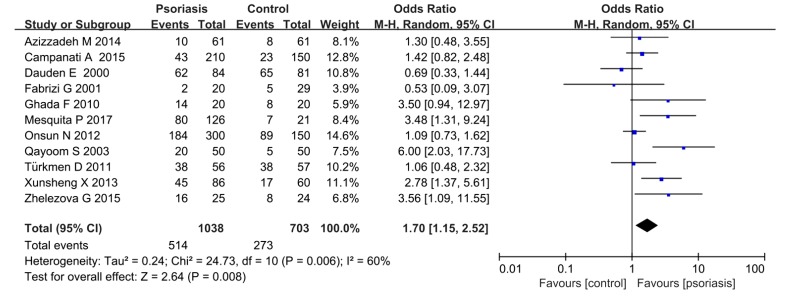
*H. pylori* infection in patients with or without psoriasis.

Among the involved cases, 1038 patients were in the psoriasis group and 703 in the control group. The Mantel–Haensze random-effects model was used because of the moderate heterogeneity (*I^2^* = 60%). The *H. pylori* infection rates in the psoriasis group were 49.5%, while they were 38.8% in the control group. The pooled OR was 1.70 (95% CI 1.15–2.52, *P =* 0.008) (Figure 2).

### 3.3. Subgroup Analysis

**Figure 3:**
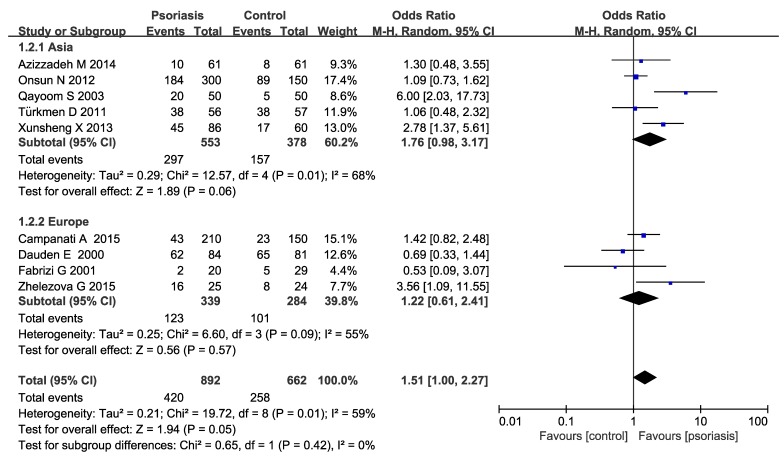
Sub-analysis according to region.

**Figure 4:**
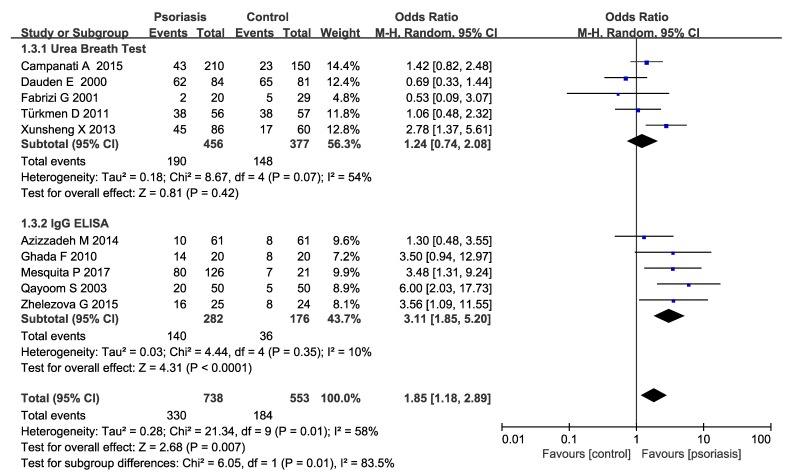
Sub-analysis according to the *H. pylori* detection methods.

**Figure 5:**
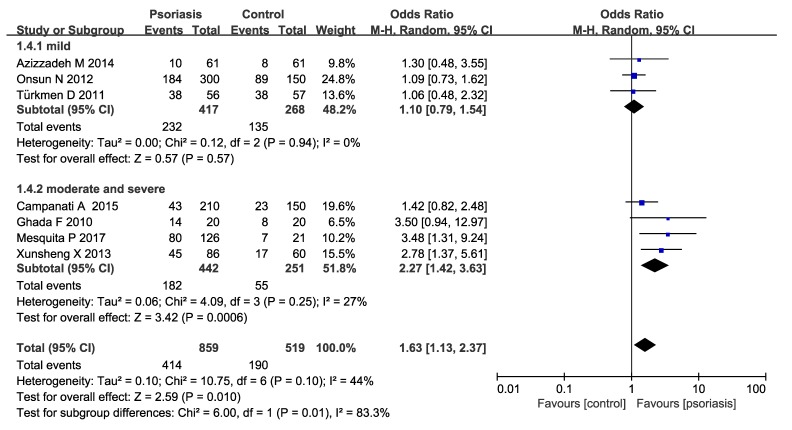
Sub-analysis according to the severity of psoriasis.

Subgroup analysis were conducted in our meta-analysis based on the following aspects: locations (Asia or Europe) (Figure 3), the methods of *H. pylori* detection (Figure 4) and the severity of psoriasis according to PASI (mean PASI ≤ 10 mild; mean PASI ≥ 10 moderate and severe; Figure 5). The subgroup analyses of locations showed that there were no significant increases in both the Asia and Europe groups (Asia pooled OR = 1.76, 95% CI 0.98–3.17, *P* = 0.06; Europe OR = 1.22 95% CI 0.61–2.41, *P* = 0.57), suggesting that region is not an influencing factor. Ghada et al. and Mesquita et al’s studies were not included in the regional subgroup analysis, since the relevant data were only from one literature. As for the methods of *H. pylori* detection, the pooled OR was 1.24 (95% CI 0.74–2.08) in urea breath test subgroup, while 3.11 (95% CI 1.85–5.20) in the IgG ELISA test group, indicating a statistically significant increase of *H. pylori* infection in IgG ELISA test group. The severity of psoriasis showed a statistically significant increase of *H. pylori* infection in moderate and severe psoriasis patients (OR=2.27; 95% CI: 1.42–3.63, *I^2^* = 27%) but not in the mild psoriasis patients (OR=1.10; 95% CI: 0.79–1.54, *I*^2^ = 0%).

### 3.4. Sensitivity Analyses and Publication Bias

By removing one study at a time, we found that none of the studies changed the pooled risk of the *H. pylori* infection rates substantially. In addition, we found no significant publication bias as assessed by the Begg’s test (*P* = 0.350) and the Egger’s test (*P* = 0.154).

## 4. Discussion

In this meta-analysis, the results showed the incidence of *H. pylori* in patients with psoriasis was 10.7% higher than that of the control group (pooled RR = 1.70, *P <* 0.01), which indicated that psoriasis might be associated with *H. pylori* infection. In the regional subgroup analysis, there were no significant increases in both Asia and Europe groups. With respect to the methods of *H. pylori* detection, there was a statistically significant increase of *H. pylori* infection in the IgG ELISA test group compared with the urea breath test group. As for the severity of psoriasis, the infection rate of *H. pylori* was higher in patients with moderate and severe psoriasis compared with the patients with mild psoriasis, indicating that *H. pylori* infection and the severity of psoriasis required more attention in clinical treatment.

The heterogeneity was moderate in this study (*I*^2^ = 60%), and regression analysis showed that different detection methods were not the source of heterogeneity. However, due to the small number of eligible literatures included in our study, it was not yet certain that different detection methods were not the sources of heterogeneity. In this meta-analysis, urea breath test, stool antigen test, and IgG ELISA test were used to detect *H. pylori* infection. It has been reported that the urea breath test was currently the best method for detecting *H. pylori*, with high accuracy and easy operation, but there might be false positive or false negative results. The stool antigen test has good sensitivity and specificity, which can detect the existing infection of *H. pylori*. The accuracy of this method is considered to be equal to that of the UBT, but currently there is a lack of reagents for the detection of monoclonal antibody with high accuracy. The IgG ELISA test can reflect *H. pylori* infection over a period of time, meanwhile, it is not affected by recent medication or local gastric lesions. At the same time, although IgG ELISA is not useful for treating patients in clinical practice, it is useful in epidemiological studies. The cause in this case is not relevant to the actual presence of the bacterium, but to the presence during the life [29]. However, *H. pylori* antibody positive cannot determine the current infection, and antibody negative cannot rule out the initial infection either [30,31,32,33]. Each of the three methods has its own advantages and disadvantages, and different methods might have a certain impact on the results of the study. It is expected that there can be uniform and accurate detection methods to provide more evidence in the future. On the other hand, subgroup analysis suggested that the severity of psoriasis might be a source of heterogeneity. *I*^2^ was significantly reduced in the mild group and the moderate and severe group (*I*^2^ = 0%, *I*^2^ = 27%, respectively). However, due to the limited literatures that conform to this study, more studies are needed to provide evidence of higher quality.

Our results indicate increased *H. pylori* infection in patients with psoriasis. The potential pathogenesis is as follows: (i) Chronic inflammation. Chronic inflammation caused by *H. pylori* might result in the release of certain cytokines and activation of immune cells, which might induce other immune-related pathologies [34]. (ii) Heat–shock protein leading to increased production of IL-6. Heat–shock protein (HSP) in *H. pylori* mediates macrophage-induced release of IL–6, which might lead to infiltration of cytokines from the gastric mucosa [35]. Previous studies have shown that psoriasis might also be mediated by IL–6 [36], which provided a possible relationship between these two diseases. (iii) Cytotoxin-associated gene A (CagA). CagA can cause host cell secretion of IL–8, and IL–8 plays an important role in the formation of psoriasis. IL–8 has chemotaxis to neutrophils and T cells. Meanwhile, it can promote generation of new blood vessels and proliferation of horniness cells [37]. Therefore, *H. pylori* infection might play a role in the pathogenesis of psoriasis vulgaris by stimulating the immune response of the organism and inducing the production of a large amount of IL–8. In addition, the pathogenesis of psoriasis might be similar to the pathogenesis of *H. pylori* infection and other skin disorders, for example, the release of vascular mediators like histamines might be the triggering factor for the development of rosacea [7].

The present study proved a significant association between psoriasis and *H. pylori* infection, in accordance with a previous study by Yong et al [38]. Yong et al did not include children in their study. In contrast to the abovementioned meta-analysis, we conducted subgroup analyses according to the region, *H. pylori* detection methods, and the severity of psoriasis to explore the corresponding relationship in depth. However, our meta-analysis also has some limitations. First, Scopus was not included in the literature retrieval, and the study finally included only 11 literatures, which might influence the credibility of the outcomes. Next, the variety of population characteristics may lead to clinical heterogeneity. Although subgroup analysis was conducted for breaking through this limitation, its influence may still be incompletely controlled. In addition, not all the studies included the severity of psoriasis, and our analysis did not consider the effect of age and gender factors. The credibility of the evidence can be improved via well-designed meta-analysis in the future, when more high-quality clinical trials are available.

## 5. Conclusions

Current evidence indicates that *H. pylori* infection is associated with psoriasis, and psoriasis patients with *H. pylori* infection may have a higher Psoriasis Area and Severity Index (PASI) score. Future studies should focalize not only on *H. pylori* infection but also on the role of gut microbiota (plus *Helicobacter*) in the pathogenesis of psoriasis.

## Figures and Tables

**Table 1:** Characteristics of studies included in the meta-analysis.

Study (Year)	Location	Study Design	Total Cases (Patients/Control)	Women (%)	Mean Age	Mean PASI	Outcomes	NOS Quality Assessment Score
Dauden E 2000 [18]	Spain (Europe)	Case-control	165 (84/81)	NA	NA	NA	Positive urea breath test	5
Campanati A 2015 [19]	Italy (Europe)	Cohort study	360 (210/150)	48.1	49.75	14.56 ± 4.35	Positive urea breath test	9
Ghada F 2010 [20]	Egypt (Africa)	Cross-sectional study	40 (20/20)	20	26–55	PASI < 15 = 3 PASI 15-25 = 7 PASI > 25 = 10	Positive *H.pylori* IgG ELISA test	9
Onsun N 2012 [21]	Turkey (Asia)	Cohort study	450 (300/150)	49	41.65	3.94 ± 4.99	Positive stool antigen test	7
Fabrizi G 2001 [22]	Italy (Europe)	Case-control	49 (20/29)	44.9	5~19	NA	Positive urea breath test	6
Zhelezova G 2015 [23]	Bulgaria (Europe)	Cross-sectional study	49 (25/24)	32	52.2	NA	Positive *H.pylori* IgG ELISA test	7
Qayoom S 2003 [24]	India (Asia)	Cross-sectional study	100 (50/50)	44	5–60	NA	Positive *H.pylori* IgG ELISA test	8
Türkmen D 2011 [25]	Turkey (Asia)	Cross-sectional study	113 (56/57)	42.9	38.4	5.89	Positive urea breath test	8
Azizzadeh M 2014 [26]	Iran (Asia)	Case-control	122 (61/61)	54	33.3	6.6 ± 3.1	Positive *H.pylori* IgG ELISA test	7
Mesquita P 2017 [27]	Brazil (South America)	Cohort study	147 (126/21)	57.9	50.48	PASI < 5 = 21 PASI 5-10 = 40 PASI > 10 = 65	Positive *H.pylori* IgG ELISA test	7
Xunsheng X 2013 [28]	China (Asia)	Case-control	146 (86/60)	41	16.5–70.5	17.42 ± 3.43	Positive urea breath test	7

***NA*** not available; ***NOS*** Newcastle-Ottawa Scale; ***IgG ELISA*** IgG enzyme linked immunosorbent.

## Supplementary Materials

The following are available online at https://www.mdpi.com/1010-660X/55/10/645/s1. Table S1: Systematic literature review search terms and strategy.
