# Supplementary material for: Helicobacter pylori Infection and Psoriasis: A Systematic Review and Meta-Analysis
Source: Medicina (Kaunas). 2019 Sep 26;55(10):645. doi: 10.3390/medicina55100645 (PMC6843633; doi:10.3390/medicina55100645)
Supplement: Supplementary file 1 [file medicina-55-00645-s001.pdf]

Title: *Helicobacter pylori* Infection and Psoriasis: A Systematic Review and Meta-Analysis

Authors: Mingyang Yu, Rongguang Zhang*, Peng Ni, Shuaiyin Chen and Guangcai Duan.

Supplementary file

Table S1. Systematic literature review search terms and strategy

**Search terms for PubMed**
#1 ("*Helicobacter pylori*"[Mesh] OR "*H. pylori*" [Title/Abstract] OR "*Campylobacter pylori*" [Title/Abstract] OR "*C. pylori*" [Title/Abstract])
#2 ("Psoriasis"[Mesh]) OR (((Psoriases[Title/Abstract]) OR Pustulosis Palmaris et Plantaris[Title/Abstract]) OR Palmoplantaris Pustulosis[Title/Abstract] OR Pustulosis of Palms and Soles[Title/Abstract] OR Pustular Psoriasis of Palms and Soles [Title/Abstract])
#1 AND #2
**Search terms for Embase**
#1 *Helicobacter pylori*/ OR *H. Pylori*. mp. OR *Campylobacter pylori*.mp. OR *C. pylori*/mp.
#2 Psoriasis.mp. OR Psoriases/ OR Pustulosis Palmaris et Plantaris.mp. OR Palmoplantaris Pustulosis/ OR Pustulosis of Palms and Soles.mp. OR Pustular Psoriasis of Palms and Soles/
#1 AND #2
**Search terms for Web of Science**
TS =(("Psoriasis" OR "Psoriases" OR "Pustulosis Palmaris et Plantaris" OR "Palmoplantaris Pustulosis" OR "Pustulosis of Palms and Soles" OR"Pustular Psoriasis of Palms and Soles") AND ("*Helicobacter pylori*" OR *"Campylobacter pylor*i" OR "*H. pylori*" OR "*C. pylori*"))
